# Effects of a Polyherbal Dietary Additive on Performance, Dietary Energetics, Carcass Traits, and Blood Metabolites of Finishing Lambs

**DOI:** 10.3390/metabo12050413

**Published:** 2022-05-03

**Authors:** Griselda Dorantes-Iturbide, José Felipe Orzuna-Orzuna, Alejandro Lara-Bueno, Luis Alberto Miranda-Romero, Germán David Mendoza-Martínez, Pedro Abel Hernández-García

**Affiliations:** 1 Posgrado en Producción Animal, Departamento de Zootecnia, Universidad Autónoma Chapingo, Texcoco CP 56230, Mexico; griseldi0993@gmail.com (G.D.-I.); jforzuna@gmail.com (J.F.O.-O.); albertomiranda@correo.chapingo.mx (L.A.M.-R.); 2 Departamento de Producción Agrícola y Animal, Unidad Xochimilco, Universidad Autónoma Metropolitana, Mexico City CP 04960, Mexico; gmendoza@correo.xoc.uam.mx; 3 Centro Universitario UAEM Amecameca, Universidad Autónoma del Estado De México, Amecameca CP 56900, Mexico; pahernandezg@uaemex.mx

**Keywords:** growth promoters, meat composition, bioactive compounds, hematological profile

## Abstract

The objective of this study was to evaluate the effects of dietary supplementation of a polyherbal additive (PA) containing hydrolyzable tannins, flavonoids, and essential oils on productive performance, dietary energetics, carcass and meat characteristics, and blood metabolites of lambs in their finishing phase. Twenty-eight Pelibuey × Katahdin lambs (20.52 ± 0.88 kg body weight (BW)) were housed in individual pens and assigned to four treatments (*n* = 7) with different doses of PA: 0 (CON), 1 (PA1), 2 (PA2), and 3 (PA3) g of PA kg^−1^ of DM for 56 days. Compared to the CON, lambs in PA1 treatment had higher average daily gain (*p* = 0.03), higher dietary energy utilization (*p* = 0.01), greater backfat thickness (*p* = 0.02), greater *Longissimus dorsi* muscle area (*p* = 0.01), and better feed conversion ratio (*p* = 0.02). PA supplementation did not affect (*p* > 0.05) dry matter intake, carcass yield, biometric measures, and meat chemical composition. All hematological and most of the blood biochemical parameters were similar in lambs of all treatments (*p* > 0.05). However, compared to the CON, lambs assigned to the PA3 treatment had lower serum urea concentration (*p* = 0.05) and higher serum albumin concentration (*p* = 0.03). In conclusion, low doses of PA could be used as a growth promoter in finishing lambs without affecting dry matter intake, carcass yield, meat chemical composition, and health status of the lambs. However, more *in vivo* research is needed to better understand the impact of bioactive compounds from PA used on productivity, metabolism, and health status of finishing lambs.

## 1. Introduction

Antibiotics have been used for several decades in ruminants as growth promoters [1]. However, the prohibition of these products in several countries has led to the search for natural products with similar effects [2]. Supplementation with polyherbal additives (PA) containing bioactive compounds could be a good strategy to improve productivity and meat quality without affecting animal health [3]. Some PA have been used to improve health [4], productive performance [5], and meat quality of finishing lambs [6]. ImmuPlus^®^ is a PA that is standardized to contain 120 g of hydrolyzable tannins per kg of product. In addition, this PA is composed of parts of several plants, such as *Ocimum sanctum* and *Andrographis paniculata*, which have high concentration of flavonoids and essential oils [7,8].

It has been reported that dietary tannins supplementation improves the efficiency of ingested nitrogen utilization and reduces energy loss due to enteric methane emissions in sheep [9]. In addition, when added to sheep diets, tannins increase the energy intake of the diet [10] and improve the total antioxidant capacity in blood serum [11]. On the other hand, it has been reported that some plants with flavonoids increase serum growth hormone levels [12], improve immune response and antioxidant enzyme activity (catalase and superoxide dismutase) in blood serum [13], and promote muscle tissue synthesis in sheep [14]. Some plants with flavonoids also increase the ruminal concentration of propionate and the abundance of rumen fiber-degrading bacteria [15]. In addition, the inclusion of flavonoid extracts in the diet modifies the expression of genes that are related to the regulation of feed intake and inflammation in finishing beef cattle [16]. Similarly, some essential oils improve the composition and function of the rumen microbiome, increase the production of volatile fatty acids in the rumen, and improve the development of the papillae of the rumen epithelium in cattle [17]. On the other hand, some essential oil mixtures increase the energy intake of diets for finishing sheep and cattle [18,19], while other essential oils improve the immune response [20] and increase the abundance of fiber-degrading microorganisms in the rumen and the digestibility of dry matter in sheep [21].

Based on the previous background, we hypothesized that dietary supplementation of a PA containing hydrolyzable tannins, flavonoids, and essential oils will benefit production performance and carcass characteristics of sheep without affecting health and meat composition. The objective of this study was to evaluate the effects of dietary inclusion of a polyherbal additive containing hydrolyzable tannins, flavonoids, and essential oils on productive performance, carcass characteristics, meat composition, blood biometry, and blood chemistry of finishing lambs fed with a high grain diet.

## 2. Results

### 2.1. Growth Performance and Dietary Energetics

Table 1 shows that lambs in the PA1 treatment were 3.5% heavier at the end of the experiment (FBW) than lambs assigned to the CON, although this difference was not significant (*p* > 0.05). An increase (*p* = 0.03) of 17% was observed for average daily gain (ADG) in the lambs of the PA1 group compared to the lambs of the CON; however, the lowest ADG was in the lambs assigned to the PA3 treatment (*p* = 0.03). Dry matter intake (DMI) and variation in DMI were similar among lambs in all treatments (*p* > 0.05). A 21% reduction (*p* = 0.02) in feed conversion ratio (FCR) was observed in lambs in the PA1 treatment compared to lambs in the CON (Table 2).

On the other hand, in the PA1 treatment lambs an increase (*p* = 0.01) of 17 and 23% was observed in the net dietary energy for maintenance (ObsNEm) and gain (ObsNEg), respectively (Table 1). Furthermore, in lambs of PA1 treatment an increase (*p* = 0.01) was noted for the relationship between observed and expected net dietary energy for maintenance (OExNEm) and for weight gain (OExNEg). However, the relationship between observed and expected DMI was lower (*p* = 0.02) in lambs of the PA1 treatment than in lambs of the CON (Table 1).

### 2.2. Carcass Traits and Carcass Biometrics

Table 2 shows the effects of treatments on carcass characteristics and morphometric measures of lambs. Dietary supplementation with PA did not affect (*p* > 0.05) hot carcass weight (HCW), hot carcass yield (HCY), cold carcass weight (CCW), cold carcass yield (CCY), and losses due to carcass cooling. However, compared to the CON, an increase (*p* ≤ 0.05) was observed for backfat thickness (BFT), *Longissimus dorsi* muscle area (LDMA), and carcass yield grade (YG) in lambs of PA1 and PA2 treatments. On the other hand, the external length of the carcass (ELC), the internal length of the carcass (ILC), chest girth (CG), leg length (LL), and carcass compactness index (CI) were similar between treatments (*p* > 0.05). However, LL tended to be lower (*p* = 0.06) for PA2 treatment than for the CON. Likewise, compared to the CON, in lambs of the PA1 treatment an increase of 10% (*p* = 0.01) was observed for the perimeter of the leg (PL).

### 2.3. Non-Carcass Components and Meat Composition

Table 3 shows that, compared to lambs in the CON, lambs assigned to the PA1 treatment had a 10.5% (*p* = 0.05) increase in stomach complex weight. However, the weight of the small intestine, large intestine, omental fat, lungs and trachea, heart, kidneys, spleen, testicles, skin, feet, and head were similar among lambs of all treatments (*p* > 0.05). On the other hand, liver weight decreased by 14% (*p* = 0.02) in lambs of the PA3 treatment with respect to the CON. Furthermore, the weight of the large intestine tended to be greater (*p* = 0.08) for the PA1 treatment than for the CON.

Table 4 shows the effects of treatments on meat chemical composition. In lambs from the PA2 treatment, protein content tended to increase (*p* = 0.09) compared to lambs from the CON. In addition, a reduction of 20% (*p* = 0.03) in the meat collagen content of lambs was observed for PA1 treatment compared to CON lambs. However, dietary supplementation with PA did not affect (*p* > 0.05) the pH, fat content, and moisture content of meat.

### 2.4. Hematological Parameters

Table 5 shows that dietary PA supplementation did not affect (*p* > 0.05) mean corpuscular volume, mean corpuscular hemoglobin, mean corpuscular hemoglobin concentration, lymphocyte, monocyte, segmented and banded neutrophil, eosinophil, and blood plasma protein contents. However, compared to CON lambs, PA3 treatment lambs showed a tendency to increase (*p* ≤ 0.10) by 7.6, 9.3, 7.6, and 23.5% the levels of hematocrit, hemoglobin, red blood cells, and blood platelets, respectively. Furthermore, compared to CON lambs, PA1 treatment lambs tended to increase by 12.3% (*p* = 0.07) the blood leukocyte concentration, while the blood basophil concentration tended to be lower (*p* = 0.06) in PA1 treatment lambs relative to CON.

### 2.5. Blood Biochemistry

Table 6 shows that dietary supplementation with increasing doses of PA did not affect the serum concentration of glucose, uric acid, cholesterol, total protein, globulin, albumin/globulin ratio, bilirubin, creatinine, lactate dehydrogenase, aspartate aminotransferase, calcium, and phosphorus (*p* > 0.05). However, a reduction of 22.9% (*p* = 0.05) was observed for serum urea concentration in the PA3 treatment lambs with respect to the CON lambs. On the other hand, compared to CON lambs, serum albumin concentration increased by 7.8% (*p* = 0.03) in PA3 treatment lambs, while serum alkaline phosphatase concentration tended to be 45% higher (*p* = 0.06) in PA2 treatment lambs relative to CON lambs.

## 3. Discussion

### 3.1. Growth Performance and Dietary Energetics

It has been reported that the effects of some PA on the productive performance of small ruminants are more effective when used at high doses than at low doses [3,22]. However, in the present study, ADG was higher in lambs supplemented with 1 g of PA than in lambs fed 2 and 3 g of the PA and the control treatment. In a similar study, Orzuna-Orzuna et al. [5] investigated the effects of other PA (0, 1, 1, 2, and 3 g kg^−1^ DM for 56 days) containing flavonoids, essential oils, and alkaloids on the productive performance of finishing lambs, and they observed higher FBW and ADG in lambs supplemented with only 1 g kg^−1^ DM of PA, but FBW and ADG decreased as the dose of PA increased, which is congruent with the results of the present study. These results suggest that low doses of PA can improve the productive performance of finishing lambs, but at high concentrations PA reduces the growth rate of lambs.

It has also been reported that the relative abundance of rumen fiber-degrading bacteria increases in response to dietary supplementation of flavonoids and essential oils [15], which could increase rumen passage rate and DMI. However, in this study, no significant changes in DMI were observed in response to dietary PA supplementation. Similar results were previously reported by Lozano-Sánchez et al. [23] in lambs supplemented with a PA (0, 5, 10, and 15 g kg^−1^ DM for 60 days) based on *Emblica officinalis* and *Ocinum sanctum* containing hydrolyzable tannins and by Orzuna-Orzuna et al. [6] in finishing lambs supplemented with PA (0, 1, 2 and 3 g kg^−1^ DM for 56 days) containing saponins, flavonoids, and polysaccharides. On the other hand, Hashemzadeh et al. [3] observed 18% higher DMI in lambs supplemented with a PA (20 g kg^−1^ DM for 48 days) based on *Rosemarinus offıcinalis*, *Cinnamomum zeylanicum*, *Curcuma longa,* and *Eugenia caryophyllata*. Together, these results suggest that PA could be more effective in stimulating DMI when administered at high doses than at low doses.

In the present study, PA supplementation did not affect the variation of DMI among individuals. In a similar study, Lozano-Sánchez et al. [23] investigated the effects of a PA (0, 5, 10, and 15 g kg^−1^ DM for 60 days) containing 12% hydrolyzable tannins on the productive performance of lambs. In that study, the variation of DMI between individuals was similar among lambs of all treatments. The absence of changes in DMI variation between individuals suggests that the PA used in the present study did not affect the welfare of the lambs.

Additionally, in this study FCR was better in lambs of PA1 treatment, suggesting that 1 g kg^−1^ DM of PA used may improve the efficiency of utilization of the feed ingested. Since no significant changes were shown in DMI, the lower FCR found in lambs of PA1 treatment could be related to the increase in the availability of ObsNEm and ObsNEg. In addition, some PA containing tannins, flavonoids, and essential oils have shown positive effects on FCR, because they increase the digestibility of ingested feed, the duodenal flux of protein and amino acids, and also reduce energy loss by enteric methane emissions [9,15,19]. Therefore, similar effects of tannin, flavonoid, and essential oil consumption as observed in our study would partially explain the better FCR in the lambs of PA1 treatment.

There is limited information on the effects of PA containing tannins, flavonoids, and essential oils on the efficiency of dietary energy utilization in finishing lambs. However, in cattle fed on a high-concentrate diet, Rivera-Méndez et al. [24] observed that dietary supplementation of hydrolyzable tannins (6 g kg^−1^ DM for 84 days) increased ObsNEm and ObsNEg by 2 and 3%, respectively. Similarly, Estrada-Angulo et al. [18] observed that, compared to CON, dietary supplementation with 150 mg d^−1^ of a mixture of essential oils (eugenol, thymol, vanillin, and limonene) increased ObsNEm and ObsNEg in lambs by 5 and 6%, respectively. Tannins, flavonoids, and essential oils were reported to reduce enteric methane production and increase ruminal propionate concentration in sheep and cattle [9,15,25], which would explain the positive effects of the PA used in the present study on ObsNEm and ObsNEg.

It was previously reported that the observed dietary net energy (NE) to expected dietary NE ratio of 1.0 indicates that ADG was consistent with the measured DMI value and with the estimated dietary NE value with tabular values [26]. Furthermore, the observed to expected dietary NE ratio greater than 1.0 suggests higher dietary energy utilization, whereas a ratio less than 1.0 indicates lower energy utilization [18,27]. In this study, the values recorded for the ratio between observed and expected dietary NE for maintenance and gain suggest that, compared to the CON, lambs in the PA1 treatment had higher efficiency of dietary energy utilization.

### 3.2. Carcass Traits, Carcass Biometrics, and Non-Carcass Components

Although it has previously been reported that dietary supplementation of tannin-containing plants can increase HCY and CCY [11], in this study HCW, HCY, CCW, CCY, ELC, ILC, CG, LL, and CI were similar in lambs of the different treatments. Similar responses were previously reported by Orzuna-Orzuna et al. [5] in lambs supplemented with increasing doses (0, 1, 2, and 3 g kg^−1^ DM for 56 days) of a PA containing flavonoids, essential oils, and alkaloids; and by Lozano-Sánchez et al. [23] in lambs supplemented with a PA (0, 5, 10, and 15 g kg^−1^ DM for 60 days) containing 12% hydrolyzable tannins.

Higher LDMA was observed in lambs from PA1 and PA2 treatments. Similarly, Redoy et al. [4] observed 16% higher LDMA in lambs supplemented with a PA (10 g kg^−1^ DM for 56 days) based on *Plantago lanceolata* and *Allium sativum* containing flavonoids and tannins. In non-ruminants, dietary supplementation of a PA based on *Tinospora cordifolia*, *Andrographis paniculata,* and *Achiranthes aspera* increased mRNA levels for the mechanistic target of rapamycin (mTOR) in skeletal muscle [28,29]. Likewise, in lambs, dietary inclusion of flavonoid-containing plants increased the diameter of muscle fibers, reduced muscle protein degradation, and increased the abundance of mTOR mRNA in skeletal muscle [14]. The mTOR pathway is among the key players involved in protein synthesis and muscle mass [30]. Similar effects of the consumption of *Tinospora cordifolia*, *Andrographis paniculata,* and flavonoids observed in our study would partially explain the increase in LDMA observed in lambs of PA1 and PA2 treatments.

In the present study, compared to lambs from the CON, lambs from PA1 and PA2 treatments had 19.9 and 15.5% higher BFT, respectively. Similarly, Liang et al. [31], using steers fed with 80% concentrate in the diet, found that dietary supplementation of a flavonoid extract (800 mg kg^−1^ DM for 80 days) increased BFT by 28%, through changes in the expression of more than 800 adipogenic and lipogenic genes. These findings suggest that some plants containing tannins, flavonoids, and essential oils increase the expression of adipogenic factors (C/EBPβ, C/EBPα, and PPARγ) in adipocytes when used at low doses but at high concentrations reduce their expression [32]. Likewise, in vitro studies [33,34] have reported that, at low doses, hydrolyzable tannins and some flavonoids (daidzein and genistein) increase adipogenesis but, at high concentrations, inhibit it. Thus, low doses of bioactive metabolites of the PA used were able to increase adipogenesis in the subcutaneous adipose tissue of lambs fed high-concentrate diets.

Except for the stomach complex and liver, in this study, supplementation of increasing doses of PA did not affect the weight of internal and external organs of finishing lambs. Similar results were previously reported by Orzuna-Orzuna et al. [6] in lambs supplemented with increasing doses of PA (0, 1, 2, and 3 g kg^−1^ DM for 56 days) based on *Acacia concinna*, *Balanites roxburghii,* and *Saccharum officinarum* and by Estrada-Angulo et al. [18] in lambs fed on high-concentrate diets and supplemented with 150 mg d^−1^ of essential oils (mixture of eugenol, thymol, limonene, and vanillin).

Lambs supplemented with 1 g PA kg^−1^ DM had higher stomach complex weight. Redoy et al. [4] observed that lambs supplemented with a PA (10 g kg^−1^ DM for 75 days) containing tannins and flavonoids had greater length and width of ruminal papillae, as well as greater thickness of rumen tunica muscularis. Also, Zhang et al. [17] reported that oregano essential oil improves the development of rumen epithelial papillae in cattle. Consequently, similar effects of the consumption of hydrolyzable tannins, flavonoids, and essential oils observed in our study would partially explain the higher stomach complex weight observed in the lambs of PA1 treatment. On the other hand, some liver diseases are known to affect the liver size and weight [35]. Therefore, the lower liver weight observed in the lambs of PA3 treatment suggests that high doses of the PA used could affect the liver health of the lambs. However, in this study PA supplementation did not affect the serum concentration of liver enzymes. In addition, although there were no significant effects, lambs on the PA3 treatment had 11.8% lower DMI than lambs on the CON. This would partially explain the lower liver weight of PA3 treatment lambs [36].

### 3.3. pH and Meat Composition

Dietary supplementation with PA did not affect the pH of lamb meat in any of the treatments. However, the pH of the meat of lambs assigned to the PA3 treatment was between 5.5 and 5.8, which is considered normal for sheep meat [37]. In a recent study, Orzuna-Orzuna et al. [11] observed that sheep meat pH decreased in response to dietary supplementation of hydrolyzable tannins. It has been reported that microbial spoilage of sheep meat increases when pH is higher than 5.8 [37]. On the other hand, low pH values reduce the growth of undesirable microorganisms in meat [38]. This suggests that the shelf life of meat from finishing lambs could increase with dietary supplementation of high doses of PA, perhaps as a consequence of the action of its bioactive metabolites. In this regard, previous studies [39,40] reported that hydrolyzable tannins and essential oils reduce a load of pathogenic microorganisms (*Escherichia coli* and *Listeria monocytogenes*) in sheep and bovine meat, which can extend the shelf life of meat.

In the present study, dietary supplementation of increasing doses of PA had no significant effect on the chemical composition of meat, indicating that the bioactive metabolites of the PA used do not affect the nutritional composition of meat from finishing lambs. Similar results were reported by Yusuf et al. [41] in goats supplemented with leaves or whole plants of *Andrographis paniculata* (15 g kg^−1^ DM for 100 days) and by Redoy et al. [4] in lambs supplemented with a PA (10 g kg^−1^ DM for 56 days) containing tannins and flavonoids. In that research, they observed that the fat, moisture, and ash content of lamb meat was similar among treatments; however, the protein content tended to increase in those lambs supplemented with PA. In the meta-analysis by Orzuna-Orzuna et al. [5], dietary supplementation with tannins was found to reduce intramuscular fat content but only in lambs less than three months of age that consumed high doses of the PA (>20 g kg^−1^ DM). In our study, the lambs were over 4 months of age, and the highest dose used was 3 g of PA with 12% tannins, which may explain the absence of changes in intramuscular fat of meat. On the other hand, lambs supplemented with 1 g PA kg^−1^ DM had lower collagen content in meat. This result could be positive because the tenderness of the meat generally increases when the collagen content decreases [6,41].

### 3.4. Blood Metabolites

During the evaluation of a new PA for domestic animals, it is important to determine its effects on the general health of the animal [5]. Blood parameters allow the detection of disorders of the hematological system and also help to identify systemic and some organ diseases [42]. In the present study, dietary supplementation of PA did not significantly affect hematological parameters. Furthermore, lambs from all treatments had hematological values within the normal range reported in the literature for healthy sheep [43]. This suggests that the bioactive metabolites of the PA used do not affect the hematological system of finishing lambs. Similar results were previously reported by Petrič et al. [44] on lambs supplemented with PA (10 g kg^−1^ DM for 77 days) containing flavonoids and essential oils and by Lozano-Sánchez et al. [40] on lambs supplemented with increasing doses of PA (0, 5, 10, and 15 g kg^−1^ DM for 60 days) based on *Emblica officinalis* and *Ocinum sanctum* containing 12% hydrolyzable tannins.

In sheep, it is possible to detect nutritional, liver, and muscle disorders using blood chemistry parameters [45]. Although, in the present study, lambs of all treatments had serum glucose concentrations above the normal range reported for healthy sheep [46], no differences between treatments were observed. This indicates that the PA used did not affect the ruminal production of propionate, which is considered the main gluconeogenic precursor in ruminants [47]. In contrast, PA consumption linearly decreased serum urea concentration. This suggests that lambs supplemented with PA had lower ruminal ammonia nitrogen concentration, perhaps due to lower ruminal degradability of the protein consumed [48], because there is a positive correlation (r = 0.55) between ruminal ammonia nitrogen concentration and serum urea concentration in ruminants [49].

Serum albumin concentration increased linearly in response to PA supplementation. However, lambs from all treatments had serum concentrations of total protein, albumin, and globulin within the range reported in the literature for healthy sheep [46,50]. This suggests that the PA used does not negatively affect nutritional status and protein catabolism in lambs fed high-concentrate diets [45,51]. On the other hand, supplementation in ruminants of some PA and essential oils reduces the serum concentration of cholesterol, triglycerides, and total lipids through changes in cellular biosynthesis and intestinal lipid absorption [52]. In the present study, PA supplementation did not affect serum cholesterol concentration, suggesting that the bioactive metabolites of the PA used did not affect intestinal absorption and cellular biosynthesis of lipids in sheep.

If used carefully and with appropriate reference intervals, serum creatinine levels serve as a sensitive marker of renal function in domestic animals [53]. For example, serum creatinine concentration above the reference interval indicates a loss of up to 75% of nephron function [54]. In the present study lambs from all treatments had serum creatinine concentrations higher than the range reported in the literature for healthy sheep [46], but there were no significant differences between treatments. This suggests that dietary supplementation with increasing doses of PA did not affect the renal health of lambs. Furthermore, according to Giannini et al. [55], the serum concentration of liver enzymes, such as alkaline phosphatase and aspartate aminotransferase, can be used as an indicator of liver function. When liver cell damage occurs, blood levels of alkaline phosphatase and aspartate aminotransferase increase [45]. In the present study, the serum concentration of liver enzymes was similar between treatments, indicating that the PA used did not affect the liver health of the lambs.

Because serum calcium and phosphorus concentration have low variability, serum levels of these two minerals are considered good indicators of ruminant nutritional status [56]. In our study, dietary supplementation of PA did not affect serum calcium and phosphorus concentrations, and lambs from all treatments had serum calcium and phosphorus levels within the normal range reported in the literature for sheep [46]. These results suggest that the PA used does not affect the mineral and nutritional status of lambs fed high-grain diets. Similarly, Orzuna-Orzuna et al. [5] investigated the effects of dietary inclusion of increasing doses of PA (0, 1, 2, and 3 g kg^−1^ of DM for 56 days) containing flavonoids, essential oils, and alkaloids in lambs fed high-grain diets. In that investigation, serum calcium and phosphorus concentrations were also unaffected by the level of PA used.

## 4. Materials and Methods

### 4.1. Experimental Location

The experiment was conducted from October to December 2021 at the Sheep Research Unit of the Universidad Autónoma Chapingo, located in Texcoco, State of Mexico, Mexico (Latitude 19°30′45″ N and Longitude 98°52′47″ W). Texcoco is located at 2250 m above sea level; the climate is temperate sub-humid with a mean annual temperature of 18.2 °C and mean annual precipitation of 665 mm [57]. All lamb care procedures were conducted following federal guidelines for the use and care of animals [58] and were approved by the Research Ethics and Bioethics Committee of the Universidad Autónoma del Estado de México.

### 4.2. Polyherbal Mixture Characteristics

The polyherbal additive (PA) used was ImmuPlus^®^ (Nuproxa S. de RL. de CV. Querétaro, Mexico), which is a standardized commercial product that contains 120 g kg^−1^ of hydrolyzable tannins. In addition, ImmuPlus^®^ is composed of the following plant parts: *Tinospora cordifolia*, *Ocimum sanctum*, *Whitania somnifera*, *Andrographis paniculata,* and *Azadirachta indica*. *T. cordifolia* contains 20.4% tannins and 2.19% flavonoids for antioxidant activity [59]; *O. sanctum* contains essential oils (24.15% camphor, 23.67% eugenol, and 13.47% eucalyptol) with antimicrobial properties [7]; *W. somnifera* contains flavonoids, tannins, and alkaloids with antimicrobial and antifungal properties [60]; *A. paniculata* contains 8.06% flavonoids and 10.22% essential oils with immunomodulatory and anti-inflammatory properties [8,61]; *A. indica* contains tannins, flavonoids, and essential oils with anti-inflammatory and antioxidant activity [62].

### 4.3. Animals, Experimental Design, and Diet Composition

Twenty-eight male Pelibuey × Katahdin lambs (20.52 ± 0.88 kg BW, 4–5 months old) were distributed using a completely randomized experimental design within one of four treatments: (1) basal diet without PA (CON); (2) PA1, CON + 1 g of PA kg^−1^ dry matter (DM); (3) PA2, CON + 2 g of PA kg^−1^ DM; and (4) PA3, CON + 3 g of PA kg^−1^ DM. Individual pens of 2 m^2^ were used to house the lambs, and each pen had an individual feeder (45 cm bunk space lamb^−1^) and an automatic drinker. The dose of 2 g of PA (ImmuPlus^®^) kg^−1^ DM was chosen based on a previous report where ingestion of this dose (2 g kg^−1^ DM) resulted in increases on ruminal total volatile fatty acids and feed efficiency in growing lambs [63]. However, the growth performance of finishing lambs and suckling calves supplemented with PA with a similar composition to the PA used in this study has shown a dose-dependent response [5,64]. Therefore, the PA concentrations used included a low dose (1 g kg^−1^ DM) and a high dose (3 g kg^−1^ DM). Seventeen days before the start of the experimental phase, all lambs were treated against internal and external parasites with 0.5 mL lamb^−1^ of ivermectin subcutaneously (Endovet^®^, Riverfarma, Labs, Mexico City, Mexico). In addition, on day 1 of the adaptation period, all lambs were vaccinated intramuscularly against *Clostridium* and *Pasteurella* (2.5 mL lamb^−1^, Bobact^®^ 8 MSD-Merck, Kenilworth, NJ, USA) and received 500,000 IU of vitamin A, 75,000 IU of vitamin D and 50 mg of vitamin E intramuscularly (Vigantol^®^, Bayer, Labs, Mexico City, Mexico).

During all days of the experimental phase, samples of offered and rejected feed were collected to determine the nutritional composition of the diets used. Samples were oven-dried at 105 °C until there was no more weight loss and subsequently ground using a Wiley mill (model 4, Arthur Thomas Co., Philadelphia, PA, USA). The samples were analyzed for the following components: dry matter, crude protein, ether extract, and ash [65]. In addition, the procedures proposed by Van Soest et al. [66] were used to determine the neutral detergent fiber and acid detergent fiber content of the feed samples. The nutritional composition of the experimental diets is shown in Table 7.

**Figure 1:**
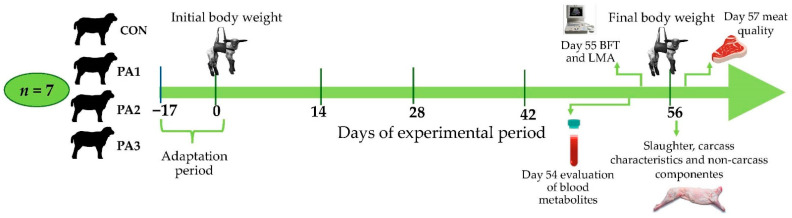
Diagram of the experimental design showing the samples taken during the experiment. CON: basal diet without polyherbal additive (PA); PA1, CON + 1 g of PA kg^−1^ dry matter (DM); PA2, CON + 2 g of PA kg^−1^ DM; and PA3, CON + 3 g of PA kg^−1^ DM. BFT: backfat thickness; LMA: *Longissimus dorsi* muscle area.

The lambs were adapted to the basal diet for 17 days, and they were included in the experimental phase, which lasted 56 days. PA was fed to the lambs through diets formulated to obtain daily weight gains of 250 g [26]. The levels of PA (1, 2, or 3 g kg^−1^ of DM) were mixed with the minor ingredients of the diet (calcium carbonate, common salt, and premix of vitamins and minerals), and then the mixture was incorporated with the rest of the ingredients until a totally mixed ration was obtained. Feed was offered to the animals every day at 7:30 a.m. and 3:30 p.m., in an amount 5% higher than the previous day’s intake to ensure *ad libitum* feed intake. Water was also available *ad libitum* throughout the experimental phase. Figure 1 shows the experimental design used and the samples taken during the experiment.

### 4.4. Calculations

On days 1 and 56 of the experimental phase, all lambs were weighed 1 h before morning feeding. Subsequently, to adjust for gastrointestinal fill, the body weight (BW) of lambs recorded on day 1 was converted to shrunk body weight (SBW) using the equation: BW × 0.96 [67]. Additionally, lambs were fasted for 12 h before recording their final body weight (FBW). Therefore, estimations for average daily gain (ADG) and net dietary energy were made based on SBW. In addition, every day the amount of feed offered and rejected was recorded to estimate dry matter intake (DMI, kg d^−1^). ADG (kg d^−1^) was calculated with the equation: (initial SBW − final SBW)/56. Feed conversion ratio (FCR) was calculated with the following equation: FCR = DMI/ADG.

According to Estrada-Angulo et al. [18,27], in productive performance trials with finishing lambs, the relationship between observed and expected net energy (NE) and the relationship between observed and expected DMI can be used to evaluate the efficiency of dietary energy utilization. Based on the observed average for ADG and SBW, and with the NE values of the diet (Table 1), expected DMI was estimated using the equation: expected DMI, kg d^−1^ = (ME/NEm) + (EG/NEg), in which ME (energy required for maintenance, expressed in Mcal d^−1^) = 0.056 × SBW^0.75^ and EG (represents energy gain, expressed in Mcal d^−1^) = ADG × 0.276 × SBW^0.75^. Meanwhile NEm (maintenance dietary energy, Mcal kg^−1^ DM) = 1.81 and NEg (gain dietary energy, Mcal kg^−1^ DM) = 1.26 were estimated based on the ingredient composition of the experimental diets [30]. Likewise, the coefficient of 0.276 was taken from NRC [68], assuming that male Pelibuey × Katahdin lambs have a mature weight of 113 kg [69].

On the other hand, using the values observed for DMI during the experiment, as well as the values of ME and EG, the observed dietary NE was calculated by means of the quadratic formula: $x=\left(-b-\sqrt{b^{2}-4\mathrm{ac}}\right)/2c$, in which x = NEm (Mcal/kg), a = −0.41 EM, b = 0.877 EM + 0.41 DMI + EG, and c = −0.877 DMI [70].

### 4.5. Carcass Traits, Carcass Biometrics, and Non-Carcass Components

On day 55 of the experimental phase, backfat thickness (BFT) and *Longissimus dorsi* muscle area (LDMA) were measured in lambs of all treatments following the methodology described by Silva et al. [71]. Prior to the evaluation, all lambs were shaved between the 12th and 13th ribs to allow skin contact with the transducer. Subsequently, a Sonovet 600 ultrasound (Medison, Inc., Cypress, CA, USA) with a 7.5 Mhz transducer was used between the 12th and 13th ribs of each lamb. In addition, the yield grade (YG) of the carcass was measured using the following equation [72]: YG = 0.4 + (10 × BFT, in cm).

Immediately after slaughter, all internal and external organs were separated from the carcass and the hot carcass weight (HCW) were recorded. All carcasses were cooled to 4 °C and 24 h later were weighed to obtain the cold carcass weight (CCW). The yield of the hot carcass (HCY) and cold carcass (CCY) was estimated using the following equations: HCY = (HCW/FBW) × 100 and CCY = (CCW/FBW) × 100, as previously reported by other authors [5,6].

Using the methodology previously described by Yañez et al. [73], morphometric measurements were performed on the cold carcass of all lambs: internal length of the carcass (ILC), external length of the carcass (ELC), chest girth (CG), leg length (LL), and perimeter of the leg (PL). In addition, the carcass compactness index (CI) was obtained using the equation CI = CCW/ILC, expressed in kg cm^−1^. Finally, the individual weights of the following organs and limbs were recorded for all lambs: head, feet, skin, liver, empty stomach complex (rumen, reticulum, omasum, and abomasum), empty small intestine (duodenum, jejunum, and ileum), empty large intestine (cecum, colon, and rectum), kidneys, heart, spleen, and lungs.

### 4.6. pH and Meat Composition

From the cold carcass (24 h post-mortem) of all lambs, samples were collected from the *Longissimus dorsi* muscle (approximately 500 g) between the 12th and 13th ribs. Subsequently, with a scalpel, fat and subcutaneous tissue were removed from all *L*. *dorsi* samples. Meat pH was measured following the methodology previously described by Orzuna-Orzuna et al. [5,6]. For this, 3 g of each muscle sample was weighed (in triplicate), and each portion of meat was homogenized with 20 mL of deionized water, using a Waring 51BL32 blender (model 700, Torrington, CT, USA). Then, a Hanna^®^ brand pH meter (Model HI 98127, Waterproof Tester, Torrington, CT, USA) was used to measure the pH of each sample in triplicate. Finally, a blender was used to grind and homogenize for 5 min the entire muscle sample from each lamb, and 150 g of sample were taken in triplicate to determine the content (g 100 g^−1^) of protein, fat, moisture, and collagen of each sample using a FOSS FoodScan™ Near-infrared spectrophotometer, as described by Anderson [74].

### 4.7. Blood Metabolites

To determine hematological and biochemical parameters, on day 54 of the experiment and prior to morning feeding (07:00 h), two 5 mL blood samples were collected from each lamb through a jugular vein puncture. The first blood sample (5 mL lamb ^−1^) was collected using tubes with BD Vacutainer^®^ K2 EDTA anticoagulant (Becton, Dickinson and Company, Franklin Lakes, NJ, USA), and then stored at 4 °C. Subsequently, the samples were analyzed on a hematology analyzer (KontroLab QS EasyVet, Michoacán, Mexico) to determine complete hemogram, hematocrit, and leukocyte count, as previously reported by Lozano-Sánchez et al. [23] and Orzuna-Orzuna et al. [5]. The second blood sample (5 mL lamb^−1^) was collected using BD Vacutainer^®^ tubes without anticoagulant (Becton, Dickinson and Company, Franklin Lakes, NJ, USA) and tubes were centrifuged at 3500 rpm for 20 min to obtain blood serum, which was stored in Eppendorf tubes at −20 °C. Subsequently, the blood serum was analyzed with an autoanalyzer (KontroLab QS EasyVet, Michoacán, Mexico) to determine the contents of glucose, urea, uric acid, cholesterol, total protein, albumin, globulins, bilirubin, creatinine, alkaline phosphatase, lactate dehydrogenase, aspartate aminotransferase, calcium, and phosphorus, as described by Orzuna-Orzuna et al. [5].

### 4.8. Statistical Analysis

All data were analyzed with the GLM procedure of SAS statistical software [75] using a completely randomized design where each lamb was considered as the experimental unit. First, the Shapiro–Wilk test was used to test the normality of the data for each variable. Subsequently, the productive performance data (DWG, BW, DMI, and FCR), the relationship between observed and expected DMI, and dietary energetics were analyzed including initial BW as a covariate, but, since this covariate was not significant (*p* > 0.05), it was eliminated from the model. Data for carcass characteristics, morphometric measurements, meat composition, blood biometry, and blood chemistry were initially analyzed including FBW as a covariate; however, in the final model FBW was removed because it was also non-significant (*p* > 0.05). The statistical model used in the data analysis was as follows: Y_ijk_ = µ + T_i_ + e_ij_, in which: µ is the value of the statistical mean, T_i_ is the fixed effect of the treatments, and e_ij_ is the standard error term. Means of treatments were compared using the Tukey test; significant differences were considered to be present when *p* ≤ 0.05, and the trend was considered to be significant when *p* > 0.05 and ≤0.10.

## 5. Conclusions

The results of this study indicate that the dietary inclusion of 1 g of PA (ImmuPlus^®^) kg^−1^ DM improves weight gain, feed conversion ratio, the efficiency of dietary energy utilization, backfat thickness, and *Longissimus dorsi* muscle area, without affecting dry matter intake, carcass yield, meat chemical composition, and health status of the lambs. Therefore, low doses of PA ImmuPlus^®^ could be used as a growth promoter in finishing lambs fed with high concentrate diets. However, further research is desirable to evaluate the effects of other doses of this PA (ImmuPlus^®^), and its individual bioactive compounds on the productivity, metabolism, and health status of sheep and other ruminants fed high-concentrate diets.

## Figures and Tables

**Table 1:** Growth performance of lambs supplemented with a polyherbal additive ^1^ during the final fattening period.

Parameters	Treatments				SEM	*p*-Value
	CON	PA1	PA2	PA3		
Initial body weight, kg	21.03	20.11	20.84	20.08	0.879	0.87
Final body weight (FBW), kg	32.96	34.11	33.73	31.83	1.191	0.50
Average daily gain (ADG), kg d^−1^	0.213 ^b^	0.250 ^a^	0.230 ^ab^	0.209 ^b^	0.015	0.03
Dry matter intake (DMI), kg d^−1^	0.906	0.848	0.865	0.799	0.048	0.13
DMI variation (%) ^2^	17.36	17.57	17.65	17.86	2.141	0.86
Feed conversion ratio (FCR), DMI/ADG	4.39 ^a^	3.47 ^b^	3.67 ^ab^	3.85 ^ab^	0.263	0.02
Observed dietary net energy, Mcal kg^−1^ of DM						
Maintenance (ObsNEm)	1.985 ^b^	2.336 ^a^	2.198 ^ab^	2.148 ^ab^	0.093	0.01
Gain (ObsNEg)	1.331 ^b^	1.639 ^a^	1.518 ^ab^	1.474 ^ab^	0.081	0.01
Observed to expected diet net energy ^3^, Mcal kg^−1^ of DM						
Maintenance (OExNEm)	1.096 ^b^	1.291 ^a^	1.214 ^ab^	1.187 ^ab^	0.051	0.01
Gain (OExNEg)	1.056 ^b^	1.301 ^a^	1.205 ^ab^	1.170 ^ab^	0.064	0.01
Observed to expected DMI	0.96 ^a^	0.79 ^b^	0.83 ^ab^	0.85 ^ab^	0.047	0.02

^1^ ImmuPlus^®^ based on *Tinospora cordifolia*, *Ocimum sanctum, Whitania somnifera, Andrographis paniculata*, and *Azadirachta indica*. ^2^ Variation in daily feed intake among individuals between days; CON—basal diet without polyherbal additive (PA); PA1—basal diet + 1 g of PA kg^−1^ of dry matter (DM); PA2—basal diet + 2 g of PA kg^−1^ of DM; PA3—basal diet + 3 g of PA kg^−1^ of DM. SEM—standard error of the treatment means; ^a, b^—means within a row with different subscripts differ when *p* ≤ 0.05; ^3^ an observed to expected dietary net energy ratio greater than 1.0 indicates higher dietary energy utilization, while a ratio less than 1.0 indicates lower energy utilization [18].

**Table 2:** Carcass traits of lambs supplemented with a polyherbal additive ^1^ during the final fattening period.

Parameter	Treatment				SEM	*p*-Value
	CON	PA1	PA2	PA3		
Hot carcass weight (HCW), kg	15.11	14.96	15.84	15.08	0.75	0.49
Hot carcass yield (HCY), %	45.16	44.23	47.06	46.04	1.23	0.28
Cold carcass weight (CCW), kg	14.50	14.27	14.73	13.91	0.63	0.51
Cold carcass yield (CCY), %	43.36	42.31	43.77	42.84	0.67	0.67
Losses due to carcass cooling, %	3.91	4.27	4.63	5.03	1.41	0.56
Backfat thickness (BFT), mm	2.31 ^b^	2.77 ^a^	2.67 ^a^	2.55 ^ab^	0.13	0.02
Muscle area *Longissimus dorsi* (LDMA), cm^2^	9.87 ^b^	11.84 ^a^	11.70 ^a^	10.70 ^ab^	0.51	0.01
Yield grade (YG)	0.39 ^b^	0.47 ^a^	0.47 ^a^	0.43 ^ab^	0.02	0.04
External length of the carcass (ELC), cm	46.71	46.57	46.00	46.14	0.86	0.56
Internal length of the carcass (ILC), cm	44.14	43.57	42.85	44.00	0.79	0.26
Chest girth (CG), cm	66.00	66.43	67.00	66.57	1.05	0.50
Length of the leg (LL), cm	29.28 *	29.57	30.71 *	29.57	0.51	0.06
Perimeter of the leg (PL), cm	34.28 ^b^	37.71 ^a^	36.43 ^ab^	36.00 ^ab^	0.93	0.01
Compactness index (CI), kg cm^−1^	0.33	0.33	0.34	0.32	0.01	0.44

^1^ ImmuPlus^®^ based on *Tinospora cordifolia*, *Ocimum sanctum, Whitania somnifera, Andrographis paniculata,* and *Azadirachta indica*. CON—basal diet without polyherbal additive (PA); PA1—basal diet + 1 g of PA kg^−1^ of dry matter (DM); PA2—basal diet + 2 g of PA kg^−1^ of DM; PA3—basal diet + 3 g of PA kg^−1^ of DM. SEM—standard error of the treatment means; ^a, b^—means within a row with different subscripts differ when *p* ≤ 0.05; *—indicates a tendency.

**Table 3:** Organ weights of lambs supplemented with a polyherbal additive ^1^ during the final fattening period.

Parameter	Treatment				SEM	*p*-Value
	CON	PA1	PA2	PA3		
Stomach complex ^2^ (empty), kg	0.955 ^b^	1.055 ^a^	1.024 ^ab^	0.937 ^ab^	0.034	0.05
Small intestine ^3^ (empty), kg	0.735	0.877	0.781	0.786	0.055	0.12
Large intestine ^4^ (empty), kg	1.082 *	1.137 *	1.078	1.056	0.063	0.08
Omental fat, kg	1.003	1.144	1.150	1.148	0.081	0.21
Lungs and trachea, kg	0.689	0.653	0.682	0.667	0.044	0.56
Heart, kg	0.167	0.176	0.170	0.169	0.005	0.11
Liver, kg	0.851 ^a^	0.761 ^ab^	0.757 ^ab^	0.730 ^b^	0.034	0.02
Kidneys, kg	0.395	0.423	0.421	0.431	0.024	0.30
Spleen, kg	0.099	0.099	0.082	0.080	0.008	0.11
Testicles, kg	0.572	0.571	0.587	0.589	0.044	0.79
Skin, kg	2.883	2.869	3.087	2.910	0.174	0.41
Head, kg	0.844	0.841	0.896	0.861	0.026	0.13
Feet, kg	1.908	1.920	2.073	2.049	0.064	0.16

^1^ ImmuPlus^®^ based on *Tinospora cordifolia*, *Ocimum sanctum, Whitania somnifera, Andrographis paniculata,* and *Azadirachta indica*; ^2^ rumen, reticulum, omasum, and abomasum; ^3^ duodenum, jejunum, and ileum; ^4^ cecum, colon, and rectum; CON—a basal diet without polyherbal additive (PA); PA1—basal diet + 1 g of PA kg^−1^ of dry matter (DM); PA2—basal diet + 2 g of PA kg^−1^ of DM; PA3—basal diet + 3 g of PA kg^−1^ of DM. SEM—standard error of the treatment means; ^a, b^—means within a row with different subscripts differ when *p* ≤ 0.05; *—indicates a tendency.

**Table 4:** Meat traits of lambs supplemented with a polyherbal additive ^1^ during the final fattening period.

Parameter	Treatment				SEM	*p*-Value
	CON	PA1	PA2	PA3		
pH	5.96	5.97	5.96	5.76	0.11	0.20
Protein, g 100 g^−1^	20.05 *	19.99	20.48 *	20.37	0.17	0.09
Fat, g 100 g^−1^	2.32	2.23	2.06	2.36	0.18	0.34
Moisture, g 100 g^−1^	75.48	75.25	74.78	75.10	0.35	0.16
Collagen, g 100 g^−1^	1.46 ^a^	1.14 ^b^	1.32 ^ab^	1.37 ^ab^	0.10	0.03

^1^ ImmuPlus^®^ based on *Tinospora cordifolia*, *Ocimum sanctum, Whitania somnifera, Andrographis paniculata,* and *Azadirachta indica*. CON—a basal diet without polyherbal additive (PA); PA1—basal diet + 1 g of PA kg^−1^ of dry matter (DM); PA2—basal diet + 2 g of PA kg^−1^ of DM; PA3—basal diet + 3 g of PA kg^−1^ of DM. SEM—standard error of the treatment means; ^a, b^—means within a row with different subscripts differ when *p* ≤ 0.05; *—indicates a tendency.

**Table 5:** Hematological profile of lambs supplemented with a polyherbal additive ^1^ during the final fattening period.

Parameter	Treatment				SEM	*p*-Value
	CON	PA1	PA2	PA3		
Hematocrit, %	30.00 *	32.34	31.86	32.28 *	0.86	0.09
Hemoglobin, g dL^−1^	8.68 *	9.23	9.13	9.49 *	0.27	0.06
Red blood cells, 10^6^ mL^−1^	7.05 *	7.41	7.48	7.59 *	0.20	0.07
Mean corpuscular volume, Fl	40.86	41.09	42.39	40.76	1.21	0.35
Mean corpuscular hemoglobin, pg	12.38	12.52	12.37	12.40	0.08	0.60
Mean corpuscular hemoglobin concentration, g dL^−1^	31.47	30.48	29.82	30.92	1.25	0.28
Platelets, 10^3^ mL^−1^	604.83 *	681.57	704.86	747.17 *	47.98	0.07
Leukocytes, 10^3^ mL^−1^	9.30 *	10.45 *	9.86	9.52	0.44	0.07
Lymphocytes, 10^3^ mL^−1^	45.86	47.14	45.67	54.28	3.80	0.13
Monocytes, 10^3^ mL^−1^	11.57	12.66	12.00	12.28	1.45	0.61
Segmented neutrophils, 10^3^ mL^−1^	38.71	38.14	41.28	36.00	4.59	0.69
Band neutrophils, 10^3^ mL^−1^	0.57	0.42	0.14	0.14	0.28	0.28
Eosinophils, 10^3^ mL^−1^	1.00	1.28	3.71	2.00	0.94	0.11
Basophils, 10^3^ mL^−1^	0.50 *	0.00 *	0.14	0.28	0.16	0.06
Plasma protein, g dL^−1^	9.31	9.27	9.50	9.58	0.15	0.20

^1^ ImmuPlus^®^ based on *Tinospora cordifolia*, *Ocimum sanctum, Whitania somnifera, Andrographis paniculata,* and *Azadirachta indica*. CON—a basal diet without polyherbal additive (PA); PA1—basal diet + 1 g of PA kg^−1^ of dry matter (DM); PA2—basal diet + 2 g of PA kg^−1^ of DM; PA3—basal diet + 3 g of PA kg^−1^ of DM. SEM—standard error of the treatment means; *—indicates a tendency.

**Table 6:** Blood serum biochemistry of lambs supplemented with a polyherbal additive ^1^ during the final fattening period.

Parameter	Treatment				SEM	*p*-Value
	CON	PA1	PA2	PA3		
Glucose, mg dL^−1^	85.43	87.71	86.71	88.57	3.54	0.50
Urea, mg dL^−1^	80.50 ^a^	69.08 ^ab^	66.83 ^ab^	62.08 ^b^	6.61	0.05
Uric acid, mg dL^−1^	0.64	0.62	0.58	0.59	0.69	0.56
Cholesterol, mg dL^−1^	62.14	57.71	64.71	61.71	5.12	0.54
Total protein, g dL^−1^	8.74	8.64	8.81	8.91	0.24	0.62
Albumin, g dL^−1^	2.67 ^b^	2.74 ^ab^	2.81 ^ab^	2.88 ^a^	0.06	0.03
Globulin, g dL^−1^	5.85	5.90	6.00	6.07	2.14	0.48
Albumin/globulin	0.44	0.46	0.45	0.47	0.02	0.31
Bilirubin, mg dL^−1^	0.15	0.18	0.19	0.18	0.02	0.16
Creatinine, mg dL^−1^	1.04	1.01	0.98	1.02	0.048	0.41
Alkaline phosphatase, UI dL^−1^	341.43 *	424.43	496.28 *	386.85	49.77	0.06
Lactate dehydrogenase, UI dL^−1^	198.86	233.28	211.43	215.14	23.90	0.31
Aspartate aminotransferase, UI dL^−1^	195.28	194.43	206.86	206.00	14.43	0.57
Calcium, mg dL^−1^	10.47	10.34	10.44	10.25	0.18	0.41
Phosphorus, mg dL^−1^	6.24	6.84	6.78	6.54	0.30	0.17

^1^ ImmuPlus^®^ based on *Tinospora cordifolia*, *Ocinum sanctum, Whitania somnifera, Andrographis paniculata,* and *Azadirachta indica*. CON—a basal diet without polyherbal additive (PA); PA1—basal diet + 1 g of PA kg^−1^ of dry matter (DM); PA2—basal diet + 2 g of PA kg^−1^ of DM; PA3—basal diet + 3 g of PA kg^−1^ of DM. SEM—standard error of the treatment means; ^a, b^—means within a row with different subscripts differ when *p* ≤ 0.05; *—indicates a tendency.

**Table 7:** Ingredients and chemical composition of the experimental diets.

Ingredients, g kg^−1^ DM	Treatments			
	CON	PA1	PA2	PA3
Oat straw	194	194	194	194
Ground sorghum	241	241	241	241
Soybean meal	81	81	81	81
Ground corn	303	302	301	300
Wheat bran	71	71	71	71
Calcium carbonate	3	3	3	3
Common salt	5	5	5	5
Mineral remix and vitamins ^a^	5	5	5	5
Bypass fat	23	23	23	23
Corn gluten	74	74	74	74
Polyherbal additive (PA) ^b^	0	1	2	3
Total	100	100	100	100
**Nutrient composition, g kg^−1^ DM**				
Dry matter	889.6	895.0	893.5	892.0
Crude protein	156.9	156.8	156.8	157.9
Ether extract	26.4	26.3	26.4	26.3
Ash	55.2	54.2	49.6	50.1
Neutral detergen fiber	260.4	274.9	270.5	275.7
Acid detergent fiber	137.5	135.0	137.9	135.7
Calculated net energy, Mcal kg^−1^				
Maintenance ^c^	1.81	1.81	1.81	1.81
Gain ^c^	1.26	1.26	1.26	1.26

CON—basal diet without polyherbal additive (PA); PA1—basal diet + 1 g of PA kg^−1^ of DM; PA2—basal diet + 2 g of PA kg^−1^ of DM; PA3—basal diet + 3 g of PA kg^−1^ of DM; ^a^ Mineral Premix and vitamins Vitasal^®^ ovino plus: calcium, 24%; phosphorus, 3%; magnesium, 2%; sodium, 8 %; chlorine, 12%; potassium, 0.5%; sulfur, 0.5%; antioxidant, 0.5%; Lasalocid, 2000 ppm; chromium, 5 ppm; manganese, 4000 ppm; iron, 2000 ppm; zinc, 5000 ppm; iodine, 100 ppm; selenium, 30 ppm; cobalt, 60 ppm; vitamin A, 500,000 UI; vitamin D, 150,000 UI; vitamin E, 1000 UI. ^b^ ImmuPlus^®^ based on *Tinospora cordifolia*, *Ocimum sanctum, Whitania somnifera, Andrographis paniculata,* and *Azadirachta indica*. ^c^ The calculation of net energy is based on NRC [26].

## Data Availability

The datasets used and analyzed during the current study are available from the corresponding author on reasonable request. The data are not publicly available due to restrictions on privacy.
